# Protein identification from electron cryomicroscopy maps by automated model building and side-chain matching

**DOI:** 10.1107/S2059798321001765

**Published:** 2021-03-30

**Authors:** Thomas C. Terwilliger, Oleg V. Sobolev, Pavel V. Afonine, Paul D. Adams, Chi-Min Ho, Xiaorun Li, Z. Hong Zhou

**Affiliations:** a New Mexico Consortium, Los Alamos, NM 87544, USA; b Bioscience Division, Los Alamos National Laboratory, Mail Stop M888, Los Alamos, NM 87545, USA; c Molecular Biophysics and Integrated Bioimaging Division, Lawrence Berkeley National Laboratory, Berkeley, CA 94720, USA; d Department of Bioengineering, University of California Berkeley, Berkeley, California, USA; e The Molecular Biology Institute, University of California, Los Angeles, CA 90095, USA; f Department of Microbiology, Immunology and Molecular Genetics, University of California, Los Angeles, CA 90095, USA; g California NanoSystems Institute, University of California, Los Angeles, CA 90095, USA; h Department of Microbiology and Immunology, Vagelos College of Physicians and Surgeons, Columbia University, New York, USA; i Hefei National Laboratory for Physical Sciences at Microscale, University of Science and Technology of China, Hefei, Anhui 230026, People’s Republic of China

**Keywords:** cryo-EM, structural biology, model building, map interpretation

## Abstract

Using single-particle electron cryo-microscopy (cryo-EM), it is possible to obtain multiple reconstructions showing the 3D structures of proteins imaged as a mixture. Here, it is shown that automatic map interpretation based on such reconstructions can be used to create atomic models of proteins as well as to match the proteins to the correct sequences and thereby to identify them. This procedure was tested using two proteins previously identified from a mixture at resolutions of 3.2 Å, as well as using 91 deposited maps with resolutions between 2 and 4.5 Å. The approach is found to be highly effective for maps obtained at resolutions of 3.5 Å and better, and to have some utility at resolutions as low as 4 Å.

## Abstract 2

A procedure for the identification of a protein in a map from electron cryomicroscopy based on automated model building and sequence assignment is presented.

## 1. Introduction   

One of the major advantages of single-particle electron cryo-microscopy (cryo-EM) as a structural biology tool is that it can be used to determine the individual structures of macromolecules present in a mixture (Verbeke *et al.*, 2020 ▸; Ho *et al.*, 2020 ▸). This capability comes from the classification process, in which each particle is assigned to a class (a view of a particular molecule), classes that share 3D information are grouped, and each group of classes is analyzed to yield an individual structure (Scheres, 2012 ▸; Sigworth, 1998 ▸; Nogales, 2016 ▸). In this classification process, different molecules (or different conformations of the same molecule) can be present as long as they can be distinguished and grouped appropriately. Recently, there have been a number of examples of reconstructions of more than one molecule or more than one state of a molecule from a mixture (Ho *et al.*, 2020 ▸; Kyrilis *et al.*, 2019 ▸; Kastritis *et al.*, 2017 ▸; Frank, 2017 ▸; Javed *et al.*, 2019 ▸; Nogales, 2016 ▸; Lyumkis, 2019 ▸).

An exciting possibility presented by this capability is the analysis of macromolecular structures with minimal or no purification (Kyrilis *et al.*, 2019 ▸; Ho *et al.*, 2020 ▸). It may eventually even be feasible to routinely carry out single-particle cryo-EM experiments using images obtained from a crude lysate or membrane extract from cells. This approach was applied to a fractionated lysate from *Chaetomium thermophilum* to yield a 4.7 Å resolution structure of fatty-acid synthase (Kastritis *et al.*, 2017 ▸) and to a lysate from *Caeno­rhabditis elegans* to yield low-resolution ribosome structures (Yi *et al.*, 2019 ▸). It has been applied to a lysate from *Plasmodium falciparum* that was enriched only by density-gradient centrifugation to yield two 3.2 Å resolution structures (Ho *et al.*, 2020 ▸). In the latter case, the proteins were identified by their patterns of side-chain density in well resolved parts of the maps. Here, we extend these approaches and present a method for the fully automatic identification of proteins in cryo-EM maps by model building and analysis of map density at side-chain positions. The methods described here are incorporated into the *CryoID* application for protein identification by cryo-EM described in Ho *et al.* (2020 ▸).

## 2. Methods   

### 2.1. Maps and sequences   

The maps and previously determined models for *P. falciparum* M18 aspartyl aminopeptidase and glutamine synthetase (Ho *et al.*, 2020 ▸) are available from entries EMD-20333 and EMD-20334, respectively, in the Electron Microscopy Data Bank (Lawson *et al.*, 2011 ▸) and entries 6pev and 6pew, respectively, in the Protein Data Bank (Berman *et al.*, 2000 ▸). The 91 deposited maps and models used in this work were chosen to represent the range of resolution from 2 to 4.5 Å and, for simplicity in analysis, to each have one unique protein chain. They were obtained from the EMDB and PDB and are listed in Supplementary Table S1 along with the *Z*-score for the correct sequence and the rank of the correct sequence (see below). Maps were automatically sharpened using the *Phenix* tool *auto_sharpen* (Terwilliger, Adams *et al.*, 2018 ▸).

### 2.2. Grouping of amino-acid side-chain types   

As was performed previously (Ho *et al.*, 2020 ▸), the 20 amino-acid side chains are grouped into six size/shape classes, making a reduced set of six amino acids (G representing VGASCTI, P, L representing LDNEQM, K representing KR, Y representing FHY, and W; see Fig. 2 in Ho *et al.*, 2020 ▸) that are used in sequence alignments.

### 2.3. Overall procedure for sequence identification   

The goal of sequence identification in this work is to choose the sequence from a large set of sequences (for example, 982 sequences in this work) that is most likely based on the density in a map. The basic idea is to build a main-chain model based on the map and then to use the density at all predicted side-chain positions in that model to generate a pseudo-sequence for the protein. This pseudo-sequence based on the map and model is then used to align each candidate sequence from the large set to the model. Finally, a likelihood score for the fit of each candidate sequence is obtained from the fit of the side chains in that candidate sequence to the density in the corresponding locations in the map.

Our procedure for sequence identification has three steps. Firstly, a model representing the unique part of a map is built (without using a sequence), where the unique part of the map is chosen automatically using any symmetry that was used in creating the map. Then, up to (by default) three of the longest chains in the model are chosen and possible alignments of each candidate sequence with these chains are examined and scored with a likelihood-based approach. The selection of which chains to build is an early step in the automatic model-building procedure, so limiting later steps to a small number of chains can substantially speed up the process. Three chains are chosen as a compromise between using just one (fastest) and using all chains (more accurate but slower). Finally, the highest scoring sequence is identified as described below.

Alignments between a candidate sequence and the three longest chains in a model are obtained in four steps. In the first step, the amino-acid side chain that best fits the map at each position in each chain is identified (Terwilliger, 2003 ▸), leading to a pseudo-sequence based on the map. To make this step rapid, a library of common rotamers and their typical densities in cryo-EM structures was used (Terwilliger, Sobolev *et al.*, 2018 ▸). To develop this library, examples were found in a set of cryo-EM structures for each rotamer of each side chain. For each example, the main-chain atoms of the example residue were superimposed on a standard template to orient the residue and its corresponding nearby density. Then, for each rotamer of each side-chain type, the average and variance of densities at grid points at and surrounding the side-chain position were calculated. These density and variance values are used as ‘expected’ side-chain density for this side chain and rotamer. In our procedure each rotamer is used without weighting by frequency. It is possible that an improved scoring could be obtained by including frequencies.

In the second alignment step, the candidate sequence and the best-fitting sequence obtained from the model and map are replaced with a reduced representation of that sequence. The reason for using a reduced representation is that many side chains have similar or even nearly identical shapes, so that they cannot readily be distinguished based on the density (for example valine and threonine side chains). Grouping those with similar shapes together can therefore reduce the number of possibilities to consider without a substantial loss of information.

In the third step, a sequence alignment (Needleman & Wunsch, 1970 ▸; Smith & Waterman, 1981 ▸) is carried out between the candidate sequence and the sequence coming from the map, yielding candidate amino acids at each position in the model. Finally, the log-likelihood score based on the map (Terwilliger, 2003 ▸) and the resulting sequences of candidate amino acids is calculated and used as the overall score for that candidate sequence.

### 2.4. Details of the procedure for sequence identification   

The sequence-identification procedure is normally carried out using the unique part of the map. The *Phenix* tool *map_symmetry* (Liebschner *et al.*, 2019 ▸) is used to find helical or point-group symmetry in the map. If any is found, a new map is created containing just the unique part of the map using the *Phenix* tool *segment_and_split_map* (Terwilliger, Sobolev *et al.*, 2018 ▸). If the keyword improper_symmetry is set (this is off by default and not used in the present work), the new map is examined to determine whether there is any local symmetry remaining. If there is, the unique part is cut out and a new map is created. The *Phenix* tool *trace_and_build* (Terwilliger *et al.*, 2020 ▸) is then used to analyze the unique part of the map and build a model. By default, only the three longest chains that can be built are kept. Once a map and model are available, each segment (a piece of chain without breaks) is compared with the map and sequences compatible with the map and model are identified.

The core of *sequence_from_map* is to generate a scoring matrix for each segment that reflects the relative probability that each possible side chain is located at each position in the segment. This probability is estimated from the map–model correlation for each side chain after examining all rotamers of the side chain and picking the one with the highest correlation (Terwilliger, 2003 ▸). A *Z*-score (the value for this side chain minus the mean for this side chain at all positions, divided by the standard deviation of this quantity) is calculated for each side chain at each position, where *Z* values less than zero are set to zero. These *Z*-scores are used as approximate minus-log-likelihood scores for side-chain probabilities and make up the scoring matrix for the segment.

A best-fitting sequence is generated for each segment by simply picking the highest scoring side chain at each position. Optionally, if score_by_residue_groups is set (as is the default and used in this work) and residue groups are defined (residue_groups in this work are the default groups VGASCTI, P, LDNEQM, KR, FHY and W), one representative of each residue group is chosen to represent all of the side chains in that group. Optionally (if the value of the parameter minimum_discrimination is defined and the parameter trim_models is set; this is not performed by default or in the current work), the scoring matrix is used to cut the model up into pieces, removing any residues where the discrimination between the most probable and least probable side chain is less than minimum_discrimination. The segments are then cut up at these points and smaller segments are created. Also optionally (if trim_models is set to some value *N*; this is not performed in this work or by default), the *N* residues at each end of each segment are removed before analysis. This option is available because residues at the ends of automatically built chains are frequently less well placed than those in the middle.

If one or more potential sequences representing the molecule in the map are provided, as is performed in this work, these sequences are scored one at a time by evaluating the optimal alignment of the sequence to the segments in the model and scoring as described next.

The alignment of a sequence to a set of segments is performed by considering all possible alignments for all segments, keeping the highest-scoring segment–sequence alignment, and then repeating the process with all remaining segments. Optionally, the alignments can be restricted to a set that uses each residue in the sequence only once (if the parameter allow_duplicates is set to False; this is not performed by default or in this work). Also optionally, sequences that are shorter than the number of residues in the model are rejected (if skip_if_too_short is set; this is also not performed by default or in this work).

The raw score for one segment–sequence alignment is given by the sum of the position-dependent minus-log-likelihood scores for all of the side chains in the alignment. This raw score is then adjusted by subtracting the mean value of raw segment–sequence alignment scores for random sequences of the same length as the sequence being considered. The random sequences are created using the residue frequency of eukaryotic amino acids (King & Jukes, 1969 ▸). This can be adjusted with the default_sequence parameter. If positive_only is set (not performed by default or in this work), negative segment–sequence alignment scores are ignored.

Once the best-fitting sequence has been identified, the side chains of the model are refitted using this sequence and a model with the fitted side chains is written out.

The average time required for a full analysis of one map was 20 h with a single processor.

## 3. Results and discussion   

### 3.1. Application of automated protein identification to previously analyzed cryo-EM maps obtained from an enriched cellular lysate   

**Figure 1:**
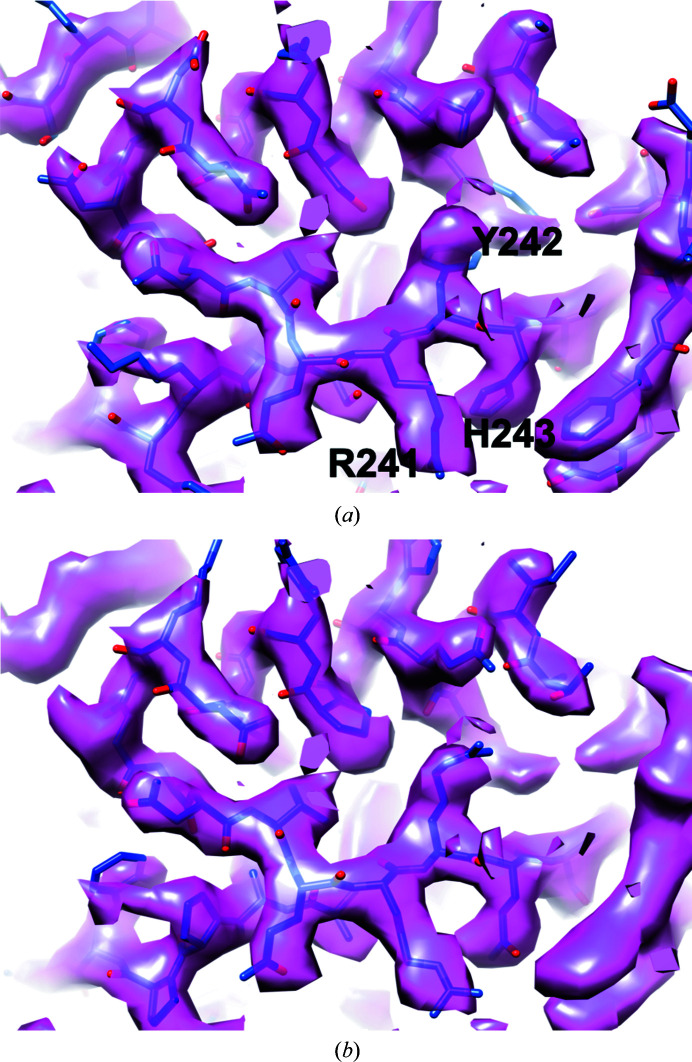
Cryo-EM map and models for glutamine synthetase from *P. falciparum*. (*a*) Deposited map and model (Ho *et al.*, 2020 ▸) with selected side chains labelled. (*b*) Deposited map and model automatically built by the *Phenix* tool *sequence_from_map*.

Our procedure for the identification of a protein from a cryo-EM map is based on the differences among the various protein side chains, which can be quite clear in a cryo-EM map at resolutions of about 3.5 Å or better. Fig. 1 ▸(*a*) shows part of a cryo-EM map for the enzyme glutamine synthetase obtained previously from an enriched cellular lysate of *P. falciparum* at a resolution of 3.2 Å (Ho *et al.*, 2020 ▸), along with the deposited model (PDB entry 6pew). Fig. 1 ▸(*b*) shows the model built automatically from this map using the *Phenix* (Liebschner *et al.*, 2019 ▸) tool *sequence_from_map* (see Section 2). Note that the sequence of the protein was not used in this model-building procedure and the side chains shown are simply the best-fitting side chains at each position. A key feature of this *Phenix* model-building approach is that the chains can be quite long, making sequence identification far more powerful for a given map quality than it would be for shorter chains. For this structure, the three chains built have 97, 56 and 54 residues, respectively.

The core algorithm in our method is side-chain identification from a map. It can be seen in Fig. 1 ▸(*b*) that the differentiation between large and small side chains is quite clear in the map at many locations along the protein backbone, and in many cases the correct side chains are placed (compare Figs. 1 ▸ *a* and 1 ▸ *b*). For each residue along a chain built into a map, we estimate the relative probability that each of the possible 20 amino acids is present at this position. This is performed using main-chain atoms in the model to predict the positions of side-chain atoms for each common rotamer of each of the 20 amino acids. The best-fitting rotamer of each type of side chain is noted, along with the map–model correlation (the correlation of expected and observed density) for that rotamer. The log-likelihood (the logarithm of the probability of observing this density for this amino acid) for each amino acid is then estimated from these map–model correlations.

This procedure is the same as we have used previously for sequence assignment in crystallographic maps (Terwilliger, 2003 ▸; Terwilliger *et al.*, 2013 ▸), except that we have generated new templates of expected density for rotamers of each side chain based on deposited cryo-EM maps (Terwilliger *et al.*, 2020 ▸). The result of this procedure is a table of probabilities for each protein chain. The table has one row for each position in the chain, and each row contains the log-likelihood scores for each of the 20 amino acids at that position.

If a chain in this analysis had no insertions or deletions, sequence alignment and ranking of one sequence relative to another would be relatively straightforward. The sequence of a protein could be compared with the table of residue log-likelihoods, and the likelihood score for a possible alignment of the sequence would be calculated simply by adding the log-likelihoods of the amino acids at each position in that alignment (Terwilliger, 2003 ▸; Terwilliger *et al.*, 2013 ▸). These likelihood scores could also be used to identify which of two sequences was more compatible with the density in the map.

Our model-building procedure, however, yields insertions or deletions in the chain tracing about once every ten amino acids (Terwilliger *et al.*, 2020 ▸). Consequently, a sequence-alignment step in which insertions and deletions are identified is necessary. In our approach, we carry out a sequence alignment for each sequence that is to be tested, identify the optimal alignment (Needleman & Wunsch, 1970 ▸; Smith & Waterman, 1981 ▸) and then use this alignment to calculate a likelihood score. The likelihood scores for the three longest segments in the model are summed to yield the overall likelihood score for a particular sequence.

In the example shown in Fig. 1 ▸, the partially purified lysate used in the analysis contained 883 proteins that could be identified by mass spectrometry (Ho *et al.*, 2020 ▸). The mean likelihood score for matching each of these 883 sequences to the automatically built model and the cryo-EM map was 3.4, with a standard deviation of 4.9. The correct sequence was readily identifiable as its likelihood score is 39.8, which is more than seven standard deviations above the mean (a *Z*-score of 7). The next-highest scoring sequence had a *Z*-score of 3.8. An analysis of the second protein previously identified in this experiment, M18 aspartyl aminopeptidase (Ho *et al.*, 2020 ▸) also yielded a conclusive identification, with a likelihood *Z*-score for the correct sequence of 8.7.

### 3.2. Application to maps from the Electron Microscopy Data Bank (EMDB)   

**Figure 2:**
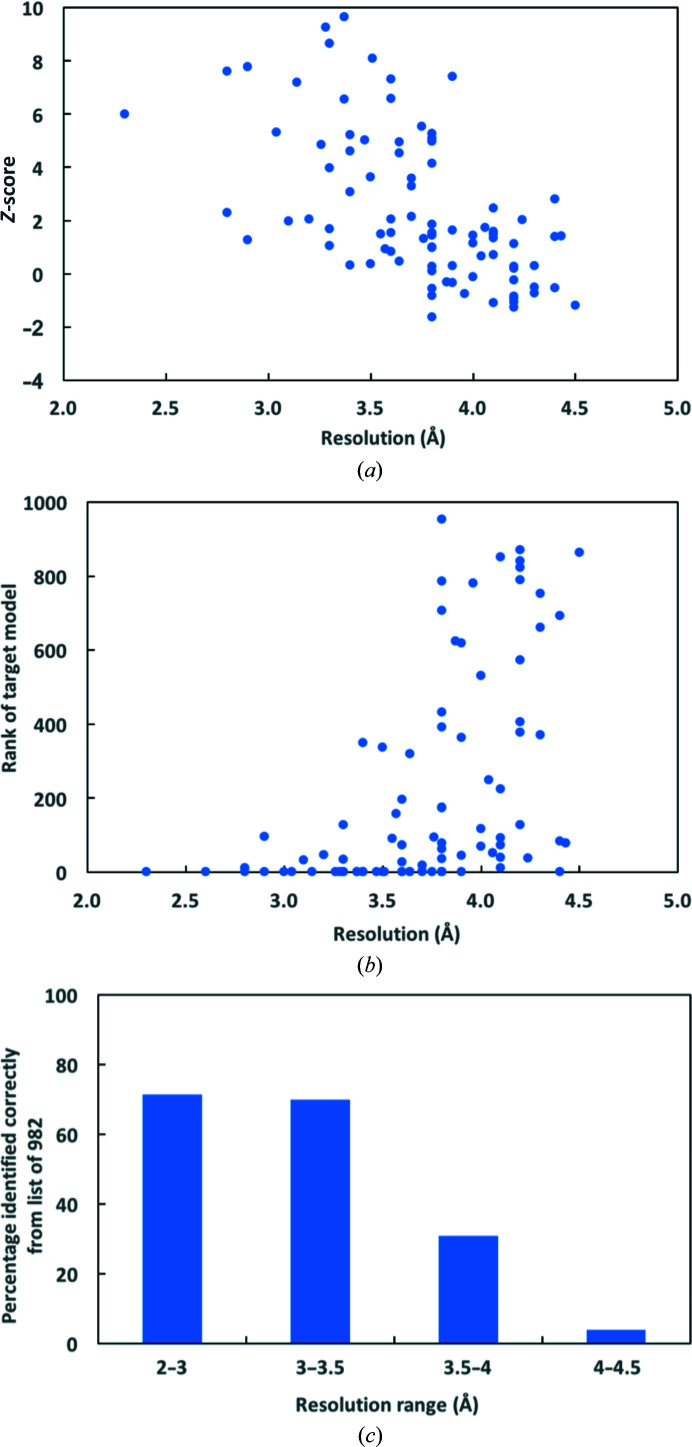
Application of automated sequence assignment to maps from the EMDB (see text). (*a*) *Z*-score of the correct sequence for each map. (*b*) Rank of the correct solution for each map. (*c*) Percentage of correct sequences that are the highest ranked in our procedure by resolution ranges.

We carried out a retrospective test to see how well this approach can work as a function of resolution of the map. We used 91 maps from the EMDB (Lawson *et al.*, 2011 ▸) with resolutions ranging from 2.2 to 4.5 Å. We analyzed each map fully automatically, building a model using each map and ranking a set of 982 sequences that included the correct sequence against the model and map. The 982 sequences consisted of 883 sequences from *P. falciparum* identified by mass spectrometry in the analysis of Ho *et al.* (2020 ▸) and 99 sequences of proteins used for model building in Terwilliger *et al.* (2020 ▸). The latter 99 sequences include 91 corresponding to the 91 maps analysed here. Fig. 2 ▸(*a*) shows the *Z*-score of the correct sequence for each map as a function of resolution. It can be seen that the *Z*-scores are strongly positive for maps in the resolution range of about 3.5 Å and better, and that they systematically become lower at worse resolutions. Fig. 2 ▸(*b*) shows the rank of the correct solution for each map. At resolutions of about 3.5 Å and better the correct sequence is generally the highest ranked. Fig. 2 ▸(*c*) illustrates the percentage of correct sequences that are the highest ranked in our procedure as a function of resolution. At resolutions of 3 Å and better the protein present in 71% of maps could be identified, and even at resolutions between 3.5 and 4 Å 30% of proteins could be identified.

### 3.3. Limitations   

The procedure developed here requires maps of sufficient quality to be interpreted automatically in terms of an atomic model and to show side chains clearly. Maps at resolutions poorer than about 4 Å typically will not be suitable for this approach, and maps that have finer resolution but have high noise will also not be likely to work well. Map interpretation at resolutions lower than about 4 Å is very challenging at present because at these resolutions side chains are often not clearly visible and main-chain conformations are difficult to identify. The present approach for sequence identification relies on an analysis of the shapes of density for side chains, so it is likely to remain limited to resolutions of about 4 Å or better, even as analysis technology improves.

In this analysis, only proteins with a single chain type were used. For proteins with multiple chains a similar procedure could be used, except that a larger number of fragments would have to be built to achieve the same sensitivity and that a step would have to be added to group fragments that match a particular sequence.

## 4. Conclusions   

In this work, we have shown that model building and identification of proteins based on cryo-EM maps at resolutions of about 3.5 Å or better can often be carried out fully automatically. These results suggest that future analyses of 3D structures of macromolecules in mixtures or lysates will be limited mainly by the ability to prepare a suitable sample and obtain high-resolution maps (*i.e.* about 3.5 Å or better) and less by subsequent analysis of the maps or identification of which protein is which.

## Supplementary Material

Supplementary Table S1. DOI: 10.1107/S2059798321001765/ir5016sup1.xlsx
